# Perceptions and concerns of emergency medicine practitioners about artificial intelligence in emergency triage management during the pandemic: a national survey-based study

**DOI:** 10.3389/fpubh.2023.1285390

**Published:** 2023-10-26

**Authors:** Erhan Ahun, Ahmet Demir, Yavuz Yiğit, Yasemin Koçer Tulgar, Meltem Doğan, David Terence Thomas, Serkan Tulgar

**Affiliations:** ^1^Department of Emergency Medicine, Sabuncuoglu Serefeddin Training and Research Hospital, Amasya, Türkiye; ^2^Department of Emergency Medicine, Faculty of Medicine, Mugla Sitki Kocman University, Mugla, Türkiye; ^3^Department of Emergency Medicine, Hamad Medical Corporation, Doha, Qatar; ^4^Blizard Institute, Barts and The London School of Medicine and Dentistry, Queen Mary University of London, London, United Kingdom; ^5^Department of Medical History and Ethics, Samsun University Faculty of Medicine, Samsun, Türkiye; ^6^Department of Medical History and Ethics, Kocaeli University Faculty of Medicine, Kocaeli, Türkiye; ^7^Department of Medical Education, Maltepe University Faculty of Medicine, Istanbul, Türkiye; ^8^Department of Pediatric Surgery, Maltepe University Faculty of Medicine, Istanbul, Türkiye; ^9^Department of Anesthesiology, Samsun University Faculty of Medicine, Samsun Training and Research Hospital, Samsun, Türkiye

**Keywords:** specialist, AI, triage, emergency, pandemic, ethics, survey, national

## Abstract

**Objective:**

There have been continuous discussions over the ethics of using AI in healthcare. We sought to identify the ethical issues and viewpoints of Turkish emergency care doctors about the use of AI during epidemic triage.

**Materials and methods:**

Ten emergency specialists were initially enlisted for this project, and their responses to open-ended questions about the ethical issues surrounding AI in the emergency room provided valuable information. A 15-question survey was created based on their input and was refined through a pilot test with 15 emergency specialty doctors. Following that, the updated survey was sent to emergency specialists via email, social media, and private email distribution.

**Results:**

167 emergency medicine specialists participated in the study, with an average age of 38.22 years and 6.79 years of professional experience. The majority agreed that AI could benefit patients (54.50%) and healthcare professionals (70.06%) in emergency department triage during pandemics. Regarding responsibility, 63.47% believed in shared responsibility between emergency medicine specialists and AI manufacturers/programmers for complications. Additionally, 79.04% of participants agreed that the responsibility for complications in AI applications varies depending on the nature of the complication. Concerns about privacy were expressed by 20.36% regarding deep learning-based applications, while 61.68% believed that anonymity protected privacy. Additionally, 70.66% of participants believed that AI systems would be as sensitive as humans in terms of non-discrimination.

**Conclusion:**

The potential advantages of deploying AI programs in emergency department triage during pandemics for patients and healthcare providers were acknowledged by emergency medicine doctors in Turkey. Nevertheless, they expressed notable ethical concerns related to the responsibility and accountability aspects of utilizing AI systems in this context.

## Introduction

Recent attention has been drawn to artificial intelligence (AI) due to its potential to enable the creation of computer systems that can replicate human intelligence and decision-making processes (1). AI has already permeated every aspect of our lives, even if we are not consciously aware of it (2). Recently AI techniques have sent vast waves across healthcare, even fueling an active discussion of whether AI doctors will eventually replace human physicians in the future (3).

Utilizing sophisticated algorithms, AI can ‘comprehend’ intricate patterns within extensive healthcare data and employ these acquired insights to enhance clinical practices. Furthermore, it can be endowed with the capability to learn and self-correct, thus refining its precision through feedback loops. An AI system aids healthcare practitioners by furnishing them with the most current medical insights from scholarly journals, textbooks, and clinical experiences, thereby ensuring optimal patient care. Additionally, AI systems are pivotal in mitigating the diagnostic and therapeutic errors intrinsic to human clinical practice (3–5). Furthermore, these AI systems extract invaluable information from extensive patient populations, facilitating real-time inferences for health risk alerts and predictions regarding health outcomes (6).

Emergency medicine, like many other medical specialties, has identified a variety of potential AI applications. Diagnosis is one of the most essential applications of AI in emergency care. In order to identify potential diagnoses, AI algorithms can examine patient data, such as symptoms, medical history, and test results, efficiently and swiftly.

AI’s role in healthcare extends significantly to include advanced patient triage capabilities. By leveraging AI algorithms to analyze patient data comprehensively, healthcare systems can effectively prioritize individuals based on the severity of their condition, ensuring that those in critical need receive immediate attention and the appropriate care interventions. This not only optimizes resource allocation but also enhances patient outcomes by minimizing delays in treatment.

In emergency medicine, AI’s influence in triage is particularly transformative. Beyond diagnosis, AI contributes to the triage process by rapidly assessing the acuity of each case. Through the analysis of various clinical indicators, such as vital signs, medical history, and presenting symptoms, AI systems can swiftly categorize patients, enabling healthcare providers to allocate resources efficiently.

By integrating AI-driven triage systems into emergency departments, healthcare facilities can improve the speed and accuracy of decision-making. AI’s ability to analyze vast datasets and adapt to real-time information empowers clinicians to make well-informed decisions, ultimately leading to more precise and timely care delivery. As a result, patients with critical conditions receive immediate attention, while those with less urgent needs are appropriately managed, resulting in enhanced overall healthcare efficiency and patient satisfaction.

The world recently endured a severe COVID-19 pandemic, during which applications of AI were also observed in the health sector. According to reports, methods exist for the analysis of radiological and laboratory results, diagnosis, patient triage in the emergency room, and the development of patient-specific treatments during a pandemic.

As with any pervasive invention, the application of AI in health has sparked ethical concerns, and these debates are ongoing in many fields (7). A few of these concerns include data privacy and security concerns, algorithmic bias, a lack of transparency, autonomy, and accountability, the dehumanization of healthcare, and economic repercussions. For AI to have the potential to improve healthcare outcomes, it must be used ethically and responsibly.

In this survey study, using the opinions of emergency medicine specialists practicing in Turkey, we sought to determine the ethical concerns and perspective of implementing AI in emergency department triage management during an epidemic.

## Materials and methods

### Study design

This survey-based study investigated the ethical perspectives of emergency specialist physicians regarding the use of artificial intelligence (AI) in the emergency department. This research was approved by the Samsun University Clinical Research Ethics Committee (SÜKAEK) (Approval No. 2022-12-12, 23/11/2022) and conducted in accordance with the ethical principles outlined in the Declaration of Helsinki. All participants gave their informed consent. Participants’ confidentiality and anonymity were maintained throughout the duration of the study. Special attention was paid to ensuring that participants did not feel compelled to participate or provide specific responses.

### Participant recruitment

Initially, ten emergency specialists were recruited to participate in the study. The research team devised and asked participants open-ended questions to gain insight into their experiences and perspectives regarding the ethical considerations of AI use in the ED.

### Survey development

Based on the responses to the open-ended questions, the research team developed a survey for gathering more specific information from the participants. The survey consisted of 15 questions and was designed to assess the emergency specialists’ ethical perspectives on the use of AI in the ED.

### Survey pilot testing

Pilot testing was conducted with 15 emergency specialist doctors. These participants were asked to provide feedback on any difficulties they had understanding the questions, issues with the appropriateness of certain questions, and grammar and spelling errors. Some changes were made to the survey to improve its clarity, readability, and comprehensiveness in response to the received feedback.

### Data collection

The revised survey was distributed to the emergency specialist doctors via email and social media tools between 05/12/2022–15/04/2023. All participants gave their informed consent. Responses were collected anonymously.

### Survey content

In the first section of the survey, participants were asked to provide descriptive information such as age, gender, and emergency medicine experience duration. On a 3-point Likert scale, participants were subsequently asked their opinions on a total of 13 questions pertaining to four major ethical topics. Before requesting opinions on each ethical topic, a thorough explanation of the ethical rule was provided. The most important ethical issues were as follows:

*Beneficence (A):* In this section, participants were asked about the beneficence of AI for triage purposes during the pandemic. The question encompassed two aspects: the usefulness of AI for patients and the usefulness of AI for physicians.

*Responsibility and accountability (B):* In this section, four questions were posed to gauge the participants’ perspectives on responsibility and accountability in the case of complications or adverse events resulting from the use of AI for triage purposes during the pandemic.

*Rights to privacy and confidentiality (C):* In this section, 5 questions on personal data protection and the right to privacy were posed to participants separately for artificial intelligence and deep learning.

*Non-Discrimination (D):* In this section, two questions were posed to ascertain the participants’ perspectives on nondiscrimination.

On a 3-point Likert scale, participants assessed a total of 20 evaluations. Through a final open-ended question, participants were also given the opportunity to express any ethical concerns not addressed in the questionnaire. The example English translation of the questionnaire is provided in Table 1.

**Table 1:** Artificial intelligence (AI) triage survey statements.

	Agree	Neutral	Disagree
A. Use of artificial intelligence for triage purposes in emergency services during COVID-19 and similar pandemics (accurate diagnosis timing, fewer complications, etc.)			
A1. It will be beneficial for the patient			
A2. It will be beneficial for the emergency medicine physician.			
B. In the event of misdiagnosis, incorrect treatment or lack of treatment, death, disability, or similar outcomes resulting from the use of artificial intelligence for triage purposes in emergency services during COVID-19 and similar pandemics.			
B1. The responsibility should only be on the practitioner			
B2. The responsibility should not only be on the practitioner, artificial intelligence and manufacturers can also be held accountable			
B3. The responsibility should only be on the artificial intelligence manufacturers or programmers.			
B4. The responsibility for complications occurring in applications varies depending on their nature			
C. The artificial intelligence program processing and retaining patients’ data in its memory for the purpose of providing ‘better guidance’.			
C1. It is a privacy violation in all circumstances			
C2. Retaining patient data in ‘Deep Learning’ based applications poses an ethical concern.			
C3. Retaining patient data in ‘Artificial Intelligence’ based systems raises ethical concerns			
C4. As long as patient data is kept under anonymous records, it does not pose a problem			
C5. The ethical dimension of storing patient data in artificial intelligence, deep learning, and similar systems during extraordinary periods like the COVID-19 pandemic can be overlooked.			
D. In terms of not engaging in discrimination, Artificial intelligence systems;			
D1. It will be as sensitive as humans, at the very least			
D2. Artificial intelligence systems, when used for triage purposes, will pose a problem in terms of ‘non-discrimination			
E. If you believe there are additional ethical issues that may arise regarding the use of artificial intelligence for triage purposes in emergency services during COVID-19 and similar pandemics, please provide your input			

To collect data for the study, a survey was developed using Google Forms and distributed via multiple social media platforms, including WhatsApp, Twitter, and Facebook. In addition, the survey was sent individually via email to groups of emergency medicine specialists. To ensure a reliable sample size, a minimum of 154 participants were required with an 80% level of confidence and a 5% margin of error, given the total population of about 2,500 specialists. The objective was to collect responses from at least 170 participants to account for the possibility of data loss.

### Statistical analysis

SPSS 16 was used for data analysis. Descriptive data were given as mean and standard deviation, and survey responses were given as frequency and percentage. *T*-test was used in the analysis of descriptive data. Categorical data were presented as counts and percentages and compared using Chi-square test or Fisher’s exact test as appropriate, with post-hoc Bonferroni adjustments to determine where the difference between groups originated. Statistical significance was accepted as *p* < 0.05.

## Results

Our survey was completed by 171 individuals within the specified time frame. Although it was clear at the outset of the survey through our social media posts that the target of the survey was emergency medicine specialists, it was discovered that 4 participants were emergency medicine residents, and as a result, only 167 participants were taken into consideration for evaluation.

**Figure 1:**
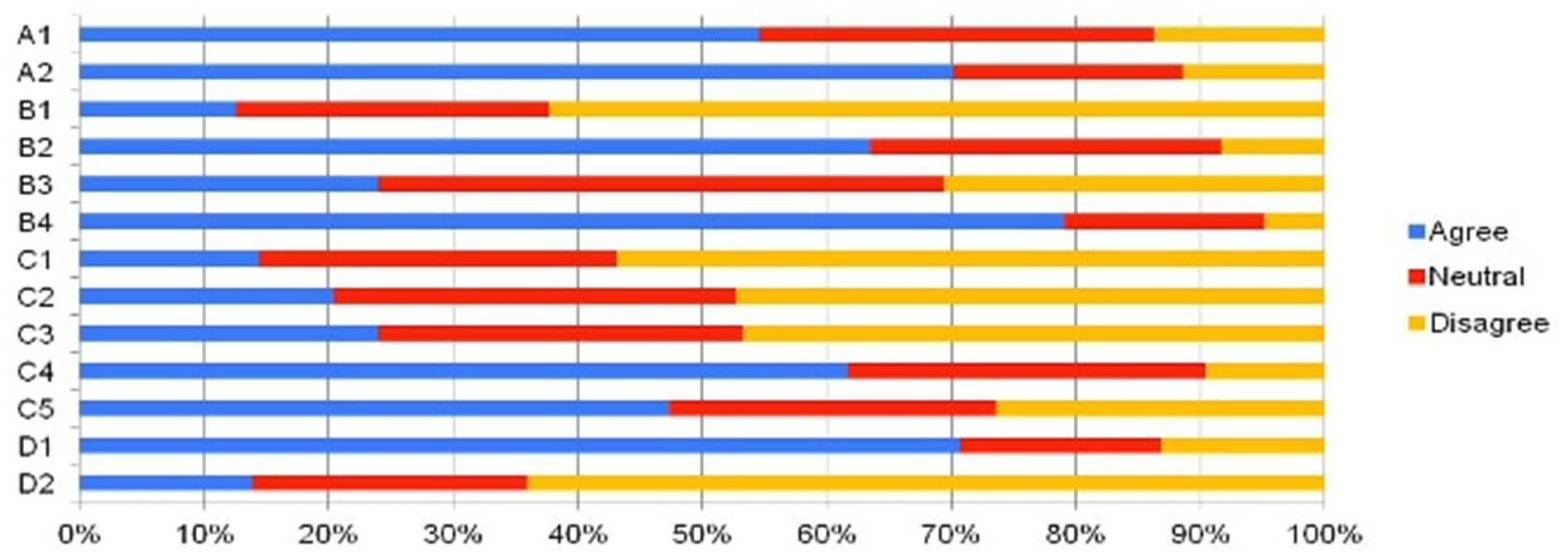
Demonstration of questionnaire results.

An overview of the survey results is shown in the Figure 1.

Participants were composed of 44 females to 123 males with an average age of 38.22 ± 6.79 years, and an average duration of professional experience of 6.79 ± 5.25 years (Table 2).

**Table 2:** The analysis of the participants’ descriptive characteristics.

Variable	Result
Age (Years)	38.32 ± 6.20
Gender (*n*, %)	
Female	44 (26.4%)
Male	123 (77.6%)
Duration of experience as an emergency medicine specialist:	
<5 y experience	66 (39.5%)
5–10 y experience	68 (40.7%)
>10y experience	33 (19.8%)

The responses to questions A1 and A2 were analyzed when evaluating the utility of using artificial intelligence for triage purposes in emergency services during COVID-19 and similar pandemics (such as accurate diagnosis time, fewer complications, etc.). 54.50% of participants believed it would be beneficial for patients, and 70.06% believed it would be beneficial for healthcare professionals.

**Figure 2:**
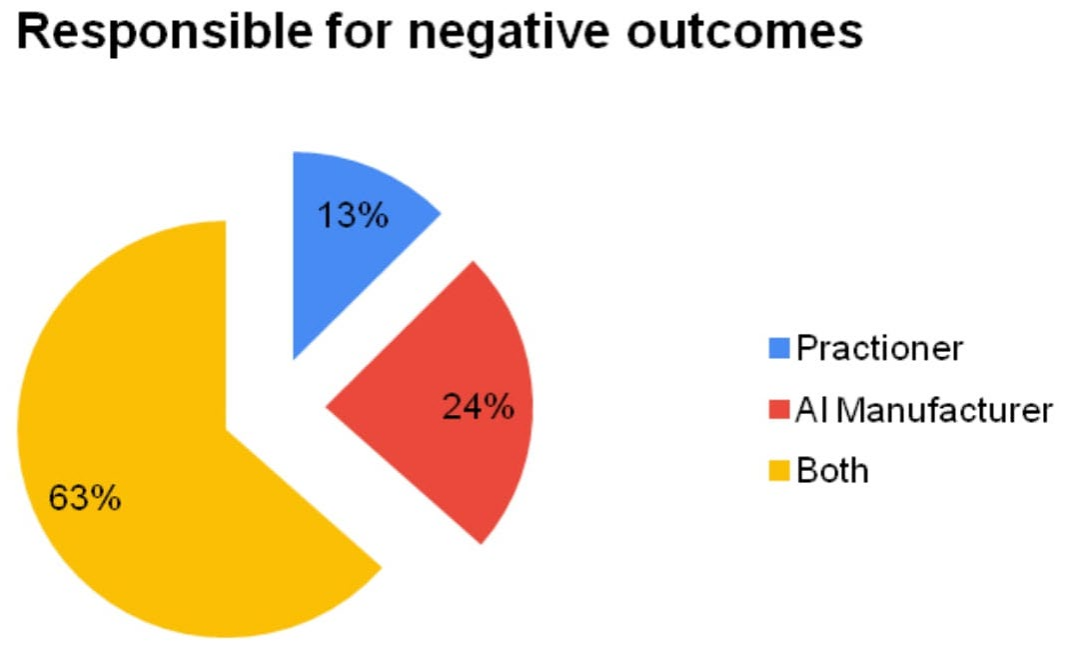
Elicitation of participants’ perceptions regarding accountability for adverse outcomes arising from the utilization of artificial intelligence (AI). Percentages have been rounded to the nearest number.

In section B, participants were asked to assess the use of artificial intelligence in pandemics from the standpoint of responsibility and accountability. Only 12.57% of participants believed that all responsibility lies with emergency medicine specialists, while 23.95% said that only artificial intelligence manufacturers and programmers should bear responsibility. The highest rate of agreement was found for the statement “The responsibility for complications occurring in applications varies depending on their nature” (79.04%). The majority, 63.47%, stated that both parties should be responsible. A demonstration of the answers given by the participants according to their agreement regarding responsibility is presented in Figure 2.

In section C, situations pertaining to the right to privacy and confidentiality were evaluated. 20.36% of participants stated that deep learning-based applications violate privacy, while 23.95% stated that AI-based applications violate privacy. 14.37% of respondents believed that these applications constitute a violation of privacy under all circumstances. Conversely, 61.68% of participants believed that there is no violation of privacy so long as the data is recorded anonymously. In addition, nearly half of the respondents (47.31%) agreed that the ethical aspect of storing patient data in artificial intelligence and similar systems could be disregarded during extraordinary events such as the COVID-19 pandemic.

In section D, opinions on the non-discrimination principle were evaluated. 70.66% of participants believe that AI-based systems would be as sensitive to non-discrimination as humans. However, only 13.37% of respondents agreed that artificial intelligence-based triage would violate the non-discrimination principle.

In section E, respondents were questioned about their perspectives on additional ethical issues not covered by the survey. Seven participants were concerned about how cultural differences might affect patient attitudes toward AI-based applications and their potential repercussions. In addition, one participant expressed concern about the elevated risk of incorrect positive/negative diagnoses as a result of the difficulties vulnerable groups face in comprehending and expressing themselves.

With the exception of two questions (*p* < 0.005), the majority of the items’ responses did not demonstrate any discernible differences based on age, gender, or years of professional experience (Table 3). There was a significant gender difference in responses to the statement that the practitioner should bear sole responsibility for negative outcomes resulting from the use of artificial intelligence in triage (question B1). The proportion of women who disagreed with this statement was higher than that of men (86.3% vs. 53.6%, *p* < 0.001) (Table 4).

**Table 3:** Assessing the relationship between participants’ demographic characteristics and their answers to questions and statistical results (*p* values).

	A1	A2	B1	B2	B3	B4	C1	C2	C3	C4	C5	D1	D2
Age (Years)	0.192	0.130	0.173	0.718	0.765	0.397	0.555	0.344	0.635	0.574	0.633	0.150	0.818
Gender	0.924	0.159	**<0.001**	0.417	0.595	0.491	0.221	0.743	0.938	0.787	0.534	0.264	0.626
Duration of experience	0.606	0.822	0.683	0.469	0.510	0.876	0.134	**0.040**	0.278	0.167	0.951	0.451	0.775

**Table 4:** Assessment of descriptive items regarding questions/statements (only statistically significant items are presented in this table).

Question no	Descriptives	Agree *n* (%)	Neutral *n* (%)	Disagree *n* (%)	*p*
B1	Female	1 (2.3%)	5 (11.3%)	38 (86.3%)*	<0.001
	Male	20 (16.2%)	37 (30%)	66 (53.6%)	
C2	<5 y experience	17 (25.7%)	19 (28.8%)	30 (45.5%)	0.040
	5–10 y experience	14 (20.6%)	27 (39.7%)	27 (39.7%)	
	>10y experience	3 (9%)	7 (21.2%)	23 (69.6%)*	

No, number; Q, question; Values are presented as number (%). *Indicates the group leading to statistically significant difference.

Another significant statistical finding was associated with question C2 of the survey, which addressed the ethical aspect of patient data storage in deep learning-based applications, specifically patient privacy. There was a higher rate of disagreement with this statement among emergency medicine specialists with over 10 years of experience compared to other experience duration groups (*p* = 0.040) (Table 4).

## Discussion

According to our findings, the majority of emergency medicine specialists in Turkey believe that using AI-based systems for triage in emergency rooms during COVID-19 and other pandemics will benefit both patients and emergency physicians. In addition, participants believe that both the artificial intelligence and the emergency medicine professional should be held accountable for any problems caused by this application, with approximately 80% agreeing that, the responsibility for complications in AI applications varies depending on their nature. A smaller proportion of participants agreed that AI’s collection of personal information violates users’ privacy, and nearly half said that this issue might be overlooked in extreme circumstances such as a pandemic. In terms of “non-discrimination,” most participants believed that artificial intelligence would be just as sensitive to this as humans, if not more so.

Triage is a critical process used in emergency medicine to effectively prioritize and manage the severity and urgency of patients’ healthcare needs. Triage is critical in quickly assessing and categorizing individuals based on the severity of their conditions and their chances of survival, especially during medical emergencies or disasters when a large influx of patients requires immediate medical attention. Medical resources can be allocated appropriately by efficiently triaging patients, ensuring that those in critical condition receive prompt care while optimizing the overall allocation of healthcare resources (1).

Triage becomes even more critical in the context of a pandemic, such as COVID-19, because the number of patients seeking medical care may exceed the available resources (2). During a pandemic, the triage process is critical for distinguishing between patients infected with the virus who require immediate medical attention and those who have mild symptoms that can be managed at home. This method ensures that resources, such as hospital beds, medical equipment, and healthcare personnel, are used as efficiently as possible (3).

Furthermore, during a pandemic, healthcare facilities may need to change their triage protocols in order to reduce the risk of infection transmission (4). Patients suspected or confirmed to have COVID-19, for example, may be triaged separately from other patients, and healthcare personnel may be required to wear personal protective equipment (PPE) to reduce their risk of exposure (6). These precautions are intended to protect both patients and healthcare workers while also slowing the spread of the virus.

Various technologies, such as telephone systems, digital scoring systems, deep learning, and AI-based systems, have been used for triage during pandemics (7–12). During the COVID-19 pandemic, AI programs have been recommended and implemented to improve patient, healthcare worker, and community safety. These AI systems consider descriptive data like age, gender, BMI, medical history, medications, contact history, and risk factors of patients who present to the emergency department. The AI systems generate an output by analyzing the patients’ current complaints, physical findings, laboratory tests, and radiological images. The analysis process employs technologies such as algorithms, machine learning, and artificial neural networks to determine probable diagnoses, urgency levels, and the severity of the patients’ conditions (13, 14). Subsequently, based on the outputs, patients can be directed to appropriate medical care units, hospitals, or facilities based on their level of urgency. Furthermore, these AI systems can aid in decision-making processes such as categorizing patients for home treatment, emergency department follow-up, or admission to a regular ward or intensive care unit (15–18).

The terms “deep learning” and “artificial intelligence” are frequently used interchangeably, but they are not the same (19). AI systems strive to imitate human learning models and demonstrate human-like intelligence. Deep learning, on the other hand, is concerned with discovering patterns and relationships in large datasets and making inferences. As a result, deep learning is only one technique in the larger field of AI. Deep learning, natural language processing, robotics, and other domains are all part of AI. While AI is used in a variety of fields, deep learning is used specifically for discovering and utilizing patterns and relationships in large datasets (20). Despite briefly mentioning the distinction between AI and deep learning in our survey, we generally preferred to use the term “AI” rather than separate the two terms. Although the use of AI in medicine appears to be promising and beneficial, it is not without ethical concerns. These include, among other things, biases, a lack of transparency, privacy, accountability and responsibility, equity, depersonalization, and autonomy (21, 22). Although this survey could have been designed in a much more comprehensive manner, we focused on the topics of beneficence responsibility and accountability, rights to privacy and confidentiality, and non-discrimination in our study. However, as the scope of survey studies grows and the time required to complete them grows, so does the participation rate. Furthermore, this is the first study to assess emergency medicine specialists’ perspectives on AI applications, and it should be viewed as a pilot ethical study focusing on a specific issue rather than a comprehensive ethical study.

Some expert opinions and survey studies have called into question the beneficence and ethical aspects of AI use in various medical fields (23, 24). However, there is currently no article that discusses the ethical implications of AI in the field of Emergency Medicine. Nonetheless, it is worth noting that studies on the ethical implications of AI use in many other areas of medicine have been published. Cobianchi et al. examined the ethical dimension of AI usage in surgical sciences in their study (using a modified Delphi process) and concluded that “the main ethical issues that arise from applying AI to surgery, relate to human agency, accountability for errors, technical robustness, privacy and data governance, transparency, diversity, non-discrimination, and fairness” (22). There are numerous recent studies discussing the ethical dimensions of AI usage in many areas of medicine, including imaging, differential diagnosis, prediction models, and decision-making, and they generally raise similar ethical concerns (25–30).

Unlike previous studies, our research is not a consensus paper reporting expert opinions or a Delphi consensus paper to address experts’ ethical concerns. Instead, we asked emergency medicine specialists who are currently or may be using AI for triage purposes during COVID-19 and similar pandemics about the ethical implications of its use. We chose to address the topics of beneficence, responsibility and accountability, privacy and confidentiality rights, and non-discrimination in our study, which focused on a single medical condition and a single purpose. We believe that, as a pilot study in the early stages of the AI era, our research will shed light on future applications. Aside from these, numerous ethical issues concerning various AI usage domains can be discussed (26).

Privacy rights and non-discrimination are prominent ethical debates in literature regarding AI. In medical ethics, the right to privacy includes not only bodily privacy but also that regarding health and personal life. As a result, individuals who are adequately informed have the right to decide how much of their information is shared (31). Within predetermined frameworks, a violation of an individual’s privacy rights may be deemed acceptable only when the benefits to the society or third parties outweigh the breach (32). While anonymizing individuals’ data before incorporating it into the system can alleviate some privacy concerns, the lack of transparency in how artificial intelligence processes data creates uncertainty about the extent to which individuals can exercise control over their own data (33). In our study, approximately 23% of participants saw retaining and subjecting data to repeated analysis within AI systems as an ethical issue, and a similar percentage saw deep learning-based systems as an ethical issue. Furthermore, 60% of participants believed that as long as the data was collected anonymously, it would not violate their privacy.

In our study, more than 70% of participants believed that AI would be as sensitive to non-discrimination as humans, while only 13% saw AI usage as an ethical concern regarding discrimination. While there is widespread agreement in the literature that AI would be more fair than humans, there are also reservations about the extent to which AI can be fair. When data generated through discriminatory thinking is fed into the system, it has the potential to perpetuate discrimination. Furthermore, the opaque decision-making mechanism of AI, which is based on established algorithms, makes identifying instances of discrimination caused by AI difficult (34, 35).

Our study has some limitations. Firstly, because it is a content-specific study conducted exclusively within the emergency medicine profession, the generalizability of our findings to a broader spectrum of healthcare practitioners may be limited. Specifically, the age distribution of the participating physicians is relatively young, which could potentially introduce bias into our results. While including older emergency physicians could offer a different perspective, it is worth noting that although emergency medicine is not a new specialty in Turkey, the recent surge in the number of graduates in the field may have contributed to the predominance of younger specialists in our study. This demographic trend, to some extent, reflects the current composition of emergency medicine professionals in the country and is a constraint beyond our control. Additionally, our survey concentrated on four specific ethical concerns chosen by the investigators, offering an in-depth exploration of these issues. However, conducting a more extensive survey, such as using the Delphi technique, might have provided a broader ethical perspective. Yet, the practical challenges of recruiting a larger participant pool could have arisen.

## Conclusion

According to our findings, emergency medicine specialists in Turkey thought that using AI programs for triage in emergency departments during pandemics could be beneficial, safe, and complication-reducing for patients and healthcare providers. Participants, however, expressed serious ethical concerns about the responsibility and accountability associated with using these systems for the stated purpose. Surprisingly, the majority of participants believed that ethical concerns about data storage and reuse could be overlooked. The perspectives of both the engineers and developers who create AI systems and the potential users, who are healthcare professionals, should be gathered more thoroughly. To develop guidelines, these perspectives should be combined with those of bioethics leaders.

## Data availability statement

The raw data supporting the conclusions of this article will be made available by the authors, without undue reservation.

## Ethics statement

The studies involving humans were approved by Samsun University Clinical Research Ethics Committee (SÜKAEK) (Approval No. 2022-12-12, 23/11/2022). The studies were conducted in accordance with the local legislation and institutional requirements. The participants provided their written informed consent to participate in this study.

## Author contributions

EA: Conceptualization, Data curation, Investigation, Methodology, Writing – original draft, Writing – review & editing. AD: Conceptualization, Data curation, Investigation, Methodology, Writing – original draft. YY: Conceptualization, Formal analysis, Methodology, Supervision, Validation, Writing – original draft, Writing – review & editing. YT: Conceptualization, Formal analysis, Investigation, Methodology, Resources, Supervision, Validation, Writing – original draft, Writing – review & editing. MD: Conceptualization, Data curation, Formal analysis, Investigation, Methodology, Writing – original draft. DT: Conceptualization, Formal analysis, Project administration, Software, Visualization, Writing – original draft. ST: Conceptualization, Data curation, Formal analysis, Investigation, Methodology, Software, Supervision, Validation, Writing – original draft, Writing – review & editing.
